# Integrating many co-splicing networks to reconstruct splicing regulatory modules

**DOI:** 10.1186/1752-0509-6-S1-S17

**Published:** 2012-07-16

**Authors:** Chao Dai, Wenyuan Li, Juan Liu, Xianghong Jasmine Zhou

**Affiliations:** 1 School of Computer, Wuhan University, Wuhan 430072, PR China; 2 Molecular and Computational Biology, Department of Biological Sciences, University of Southern California, Los Angeles, CA 90089, USA

## Abstract

**Background:**

Alternative splicing is a ubiquitous gene regulatory mechanism that dramatically increases the complexity of the proteome. However, the mechanism for regulating alternative splicing is poorly understood, and study of coordinated splicing regulation has been limited to individual cases. To study genome-wide splicing regulation, we integrate many human RNA-seq datasets to identify splicing module, which we define as a set of cassette exons co-regulated by the same splicing factors.

**Results:**

We have designed a tensor-based approach to identify co-splicing clusters that appear frequently across multiple conditions, thus very likely to represent splicing modules - a unit in the splicing regulatory network. In particular, we model each RNA-seq dataset as a co-splicing network, where the nodes represent exons and the edges are weighted by the correlations between exon inclusion rate profiles. We apply our tensor-based method to the 38 co-splicing networks derived from human RNA-seq datasets and indentify an atlas of frequent co-splicing clusters. We demonstrate that these identified clusters represent potential splicing modules by validating against four biological knowledge databases. The likelihood that a frequent co-splicing cluster is biologically meaningful increases with its recurrence across multiple datasets, highlighting the importance of the integrative approach.

**Conclusions:**

Co-splicing clusters reveal novel functional groups which cannot be identified by co-expression clusters, particularly they can grant new insights into functions associated with post-transcriptional regulation, and the same exons can dynamically participate in different pathways depending on different conditions and different other exons that are co-spliced. We propose that by identifying splicing module, a unit in the splicing regulatory network can serve as an important step to decipher the splicing code.

## Background

Alternative splicing provides an important means for generating proteomic diversity. Recent estimates indicate that nearly 95% of human multi-exon genes are alternatively spliced [1]. The mechanism for regulating alternative splicing is still poorly understood, and its complexity attributes to the combinatorial regulation of many factors, e.g. splicing factors, cis-regulatory elements, and RNA secondary structure [2,3]. A fundamental task of alternative splicing research is to decipher splicing code and understand the mechanism of how an exon is alternatively spliced in tissue-specific manner.

A central concept in transcription regulation is the *transcription module*, defined as a set of genes that are co-regulated by the same transcription factor(s). Analogously, such coordinated regulation also occurs at the splicing level [4-6]. For example, the splicing factor *Nova *regulates exon splicing of a set of genes that shape the synapse [6]. However, the study of such coordinated splicing regulation has thus far been limited to individual cases [5-9]. In this paper, we define a *splicing module *as a set of exons that are regulated by the same splicing factors. The exons in a splicing module can belong to different genes, but they exhibit correlated splicing patterns (in terms of being included or excluded in their respective transcripts) across different conditions, thus form an exon co-splicing cluster.

The recent development of RNA-seq technology provides a revolutionary tool to study alternative splicing. From each RNA-seq dataset, we can derive not only the expression levels of genes, but also those of exons and transcripts (i.e., splicing isoforms). Given an RNA-seq dataset containing a set of samples, we can calculate the inclusion rate of each exon (In this study we only consider cassette exons, which are common in alternative splicing events. Henceforth, the term "exon" always means "cassette exons".) In every sample, as the ratio between its expression level and that of the host gene. A recent study provided a nice example of studying splicing regulatory relationships using a network of exon-exon, exon-gene, and gene-gene links [10]. Here, we construct from each RNA-seq dataset a *weighted co-splicing network *where the nodes represent exons and the edge weights are correlations between the inclusion rates of two exons across all samples in the dataset. While directly comparing the inclusion rates for the same exon in different datasets could be biased by platforms and protocols, the *correlations *between inclusion rates for a given exon pair are comparable across datasets. From a series of RNA-seq datasets, we can therefore derive a series of co-splicing networks, which can be subjected to comparative network analysis and provide an effective way to integrate a large number of RNA-seq experiments conducted in different laboratories and using different technology platforms.

A heavy subgraph in a weighted co-splicing network represents a set of exons that are highly correlated in their inclusion rate profiles; i.e., they are co-spliced. A set of exons which *frequently *form a heavy subgraph in multiple datasets are likely to be regulated by the same splicing factors, and thus form a splicing module. We call such patterns *frequent co-splicing clusters*. Due to the enhanced signal to noise separation, frequent clusters are more robust and are more likely to be regulated by the same splicing factors (thus more likely to represent splicing modules) than those heavy subgraphs derived from a single dataset. In our previous research [11], we showed that the likelihood for a gene co-expression cluster to be a transcription module increases significantly with the recurrence of clusters in multiple datasets. A similar principle applies to splicing modules.

**Figure 1:**
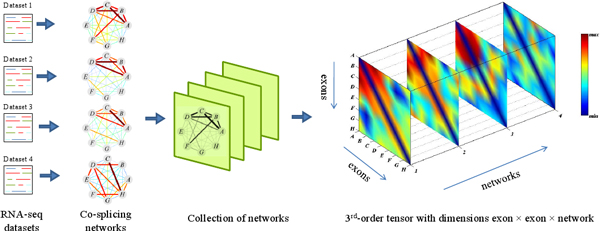
**Illustration of the 3**^**rd**^**-order tensor representation of a collection of networks**. A collection of co-splicing networks can be "stacked" into a third-order tensor such that each slice represents the adjacency matrix of one network. The weights of edges in the co-splicing networks and their corresponding entries in the tensor are color-coded according to the scale to the right of the figure. After reordering the tensor by the exon and network membership vectors, a frequent co-splicing cluster (colored in red) emerges in the top-left corner. It is composed of exons *A*, *B*, *C*, *D *which are heavily interconnected in networks 1, 2, 3.

In this paper, we adopt our recently developed tensor-based approach to find the heavy subgraph that frequently occur in multiple weighted networks [12]. Our goal here is to identify co-spliced exon clusters that frequently occur across multiple weighted co-splicing networks. A co-splicing network of *n *nodes (exons) can be represented as an *n *× *n *adjacency matrix *A*, where element *a*_*ij *_is the weight of the edge between nodes *i *and *j*. This weight represents the correlation between the two exons' inclusion rate profiles. Given *m *co-splicing networks with the same *n *nodes but different edge weights, we can represent the whole system as a 3^rd^-order tensor (or 3-dimensional array) of size *n *× *n *× *m*. An element *a*_*ijk *_of the tensor is the weight of the edge between nodes *i *and *j *in the *k*^th ^network (Figure 1). A co-splicing cluster appears as a heavy subgraph in the co-splicing network, which in turn corresponds to a heavy region in the adjacency matrix. A *frequent *co-splicing cluster is one that appears in multiple datasets, and appears as a heavy region of the tensor (Figure 1). Thus, the problem of identifying frequent co-splicing clusters can intuitively be formulated as the problem of identifying heavy subtensors in a tensor. By representing networks and formulating the problem in this tensor form, we gain access to a wealth of established optimization methods for multidimensional arrays. Reformulating a discrete graph discovery problem as a continuous optimization problem is a longstanding tradition in graph theory. There are many successful examples, such as using a Hopfield neural network to solve the traveling salesman problem [13] and applying the Motzkin-Straus theorem to the clique-finding problem [14]. Moreover, when a graph-based pattern mining problem is transformed into a continuous optimization problem, it becomes easy to incorporate constraints representing prior knowledge. Finally, advanced continuous optimization techniques require very few *ad hoc *parameters, in contrast with most heuristic graph combinatorial algorithms.

We applied our tensor algorithm to 38 weighted exon co-splicing networks derived from human RNA-seq datasets. We identified an atlas of frequent co-splicing clusters and validated them against four biological knowledge bases: Gene Ontology annotations, RNA-binding motif database, 191 ENCODE genome-wide ChIP-seq profiles, and protein complex database. We demonstrate that the likelihood for an exon cluster to be biologically meaningful increases with its recurrence across multiple datasets, highlighting the benefit of the integrative approach. Moreover, we show that co-splicing clusters can reveal novel functional groups that cannot be identified by co-expression clusters. Finally, we show that the same exons can dynamically participate in different pathways, depending on different conditions and different other exons that are co-spliced.

## Results

We identified 38 human RNA-seq datasets from the NCBI Sequence Read Archive (http://www.ncbi.nlm.nih.gov/sra) (see additional file 1: Section S6 to get details of these datasets), each with at least 6 samples providing transcriptome profiling under multiple experimental conditions, such as diverse tissues or various diseases. For each dataset, we used Tophat [15] tool to map short reads to the *hg19 *reference genome and applied the transcript assembly tool Cufflinks [16] to estimate expressions for all transcripts with known UCSC transcript annotations [17]. We calculated the inclusion rate of each exon, as the ratio between its expression (the sum of FPKM (FPKM stands for "Fragments Per Kilobase of exon per Million fragments mapped", as defined in [16].) over all transcripts that cover the exon) and the host gene's expression (the sum of FPKM over all transcripts of the gene). It is worth noting that in RNA-seq experiments, a gene expression with low FPKM is usually not precisely estimated because the number of reads mapped to the gene is quite small. In order to work with reasonably accurate estimates of exon inclusion rates, as pointed out by [18], we calculated inclusion rates only for those genes whose expressions are above 80^th ^percentile across at least 6 samples. This criterion resulted in inclusion rate profiles for 16,024 exons covering 9,532 genes. Based on these profiles, we constructed an exon co-splicing network from each RNA-seq dataset by using Pearson's correlation between exons' inclusion rate profiles. Details of data processing refer to additional file 1 (Section S5).

We applied our method to 38 RNA-seq datasets generated under various experimental conditions. Adopting the empirical criteria of "heaviness" ≥ 0.4 and cluster size ≥5 exons, we identified 7,194/3,104/1,422/594 co-splicing clusters with recurrences ≥3/4/5/6.

### Frequent co-splicing clusters are likely to represent functional modules, splicing modules, transcriptional modules, and protein complexes

To assess the biological significance of the identified patterns, we evaluate the extent to which these exon clusters represent functional modules, splicing modules, transcriptional regulatory modules, and protein complexes. Due to the difference of background "gene" numbers, we set different p-value thresholds for significance test.

#### Functional analysis

**Figure 2:**
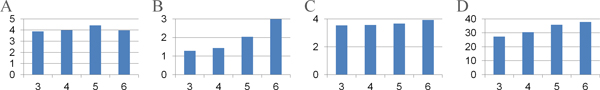
**Evaluation of the functional, splicing, transcriptional, and protein complex homogeneity of co-splicing clusters with different recurrences**. Four types of databases are used: **(A) **Gene Ontology for functional enrichment, **(B) **SpliceAid2 database for splicing enrichment, **(C) **ENCODE database for transcriptional and epigenetic enrichment, and **(D) **CORUM database for protein complex enrichment. The *x*-axis is network recurrence and *y*-axis is enrichment fold ratio, calculated by dividing the percentage of enriched clusters by the percentage of enriched random clusters.

We evaluated the functional homogeneity of the host genes in an exon cluster using Gene Ontology (GO) annotations. To ensure the specificity of GO terms, we filtered out general GO terms associated with *>*300 genes. If the host genes of exons in a cluster are statistically enriched in a GO term with *p*-value < 1E-4 (based on the hypergeometric test), we declare the exon cluster to be functionally homogeneous. We found that 23.3% of clusters appearing in ≥3 datasets are functionally homogenous, compared to only 6.0% of randomly generated clusters with the same sizes. This enrichment fold ratio of 3.9 between real and random patterns demonstrates the strong biological relevance of the identified patterns. Furthermore, the enrichment fold ratio increases with its recurrence of FSCs (shown in Figure 2A). For example, when the FSCs are required to recur in at least 5 datasets, their enrichment fold ratio compared to random patterns increases to 4.4, confirming the benefits of the integrative analysis of *multiple *RNA-seq datasets in improving the quality of detected patterns. Functionally homogenous clusters cover a wide range of post-transcriptional associated GO terms, such as "RNA splicing", "ribonucleoprotein binding", "heterogeneous nuclear ribonucleoprotein complex", "negative regulation of transcription from RNA polymerase II promoter", and "cellular protein localization".

#### Splicing regulatory analysis

By construction, the exons in our identified co-splicing clusters have highly correlated inclusion rate profiles across different experimental conditions. Clusters meeting this criterion are likely to consist of exons co-regulated by the same splicing factors. It has been shown that splicing factors can affect alternative splicing by interacting with cis-regulatory elements in a position-dependent manner [19]. We collected the experimental RNA target motifs (2220 RNA binding sites) of 62 splicing factors from the SpliceAid2 database [20]. To identify possible splicing factors associated with a co-splicing cluster, for each exon of a co-splicing cluster, we retrieved the internal exon region and its 50bp flanking intron region which are enriched in the motifs of those 62 splicing factors by performing BLAST search (E-score < 0.001). If the exons of a cluster are highly enriched in the targets of a splicing factor, we consider the cluster to be "splicing homogeneous". Although the collection of known splicing motifs is very limited, at the *p*-value < 0.05 level (based on hypergeometric test), we still observed that 4.9% clusters with ≥5 exons and ≥6 recurrences are splicing homogenous, compared to 1.6% of randomly generated patterns with the same size distribution. Its enrichment fold ratio is 3.0. Performing the same analysis for less frequent clusters, we found that as the recurrence increases, so does the enrichment fold ratio (Figure 2B). The five most frequently enriched splicing factors are *hnRNP E2*, *9G8*, *hnRNP U*, *SRp75 *and *SRp30c*. *hnRNP E2 *and *hnRNP U *both belong to heterogeneous nuclear ribonu-cleoprotein family, which generally suppress splicing through binding to exonic splicing silencer [2]. Studies show that *hnRNP E2 *can repress exon usage when present at high levels in vitro [21], and *hnRNP U *bind pre-mRNA as well as nuclear mRNA and play an important role in processing and transport of mRNA [22]. *9G8*, *SRp75 *and *SRp30c *all belong to the *SR *family of splicing regulators. *9G8 *protein excluding other *SR *factors can rescue the splicing activity of a *9G8*-depleted nuclear extract, indicating 9G8 plays a crucial role in splicing [23]. *SRp75 *is present in messenger ribonucleoproteins in both cycling and differentiated cells, and shuttles between nucleus and cytoplasm, implicating its widespread roles in splicing regulation [24]. *SRp30c *can function as a repressor of 3' splice site utilization and *SRp30c-CE9 *interaction may contribute to the control of *hnRNP A1 *alternative splicing [25].

We found that some splicing factors tend to co-bind to the cis-regulatory regions of exons in a co-splicing cluster, suggesting the combinatorial regulation of those splicing factors. Trans-acting *SR *proteins *Tra2α *and *SRp30c *are simultaneously enriched in 18 clusters (with recurrence ≥3), whose major functions (by GO term enrichment) are related to post-transcriptional regulation, such as "ribonucleoprotein binding" (*p*-value = 2.11E-5), "nuclear mRNA splicing, via spliceosome" (*p*-value = 7.66E-5), "RNA export from nucleus" (*p*-value = 4.81E-5), and "translational initiation" (*p*-value = 2.48E-5). Current study indeed shows that there is a cooperative interaction between *Tra2α *and *SRp30c *in exonic splicing enhancer dependent *GnRG *pre-mRNA splicing [26]. Splicing regulators *SRp20*, *SRp30c *and *SRp75 *are simultaneously enriched in 2 clusters (with recurrence ≥3), which are also associated with post-transcriptional regulation. For example, "RNA splicing" (*p*-value = 3.25E-6), "translation initiation factor activity" (*p*-value = 7.42E-5), and "eukaryotic translation initiation factor 3 complex" (*p*-value = 2.17E-4). Our results suggest that combinatorial splicing regulation can occur in post-transcriptional processes.

#### Transcriptional and epigenomic analysis

To evaluate how co-splicing is affected by transcriptional regulation, we used 191 ChIP-seq profiles generated by the Encyclopedia of DNA Elements (ENCODE) consortium [27]. This dataset includes the genome-wide bindings of 40 transcription factors (TF), 9 histone modification marks, and 3 other markers (DNase, FAIRE, and DNA methylation) on 25 different cell lines. For a detailed description of the signal extraction procedure, see the additional file 1 (Section S7). If the host genes of an exon cluster are highly enriched in the targets of any regulatory factor, we consider the cluster to be "transcription homogenous". At the significance level *p-*value *<*0.01, 74.9% clusters with recurrences ≥3 are transcription homogenous, compared to only 21.2% of randomly generated clusters with the same sizes. As expected, the enrichment fold ratio increases with recurrence (Figure 2C). This result suggests a strong association between transcription and splicing. The four most frequently enriched regulatory factors are *TAF8*, *GABP*, *FOS *and *NFYB*. *TAF8 *is a subunit of transcription initiation factor *TFIID*, which is required for accurate and regulated initiation by RNA polymerase II [28]. As an ETS transcription factor, *GABP *plays a key role in regulating genes which are intimately involved in cell cycle control, protein synthesis and cellular metabolism [29]. *FOS *can dimerise with *c-Jun *to form *AP-1 *transcription factor, which upregulates transcription of a wide range of genes involved in proliferation and differentiation to defense against invasion and cell damage [30]. *NFYB *is a subunit of an ubiquitous heteromeric transcription factor *NF-Y, *which regulates 30% of mammalian promoters [31].

#### Protein complex analysis

We evaluate the extent to which host genes of our identified exon clusters are protein complexes by using the Comprehensive Resource of Mammalian protein complexes database (CORUM, September 2009 version) [32]. At the significance level *p-*value *<*0.05, 18.1% of co-splicing clusters with recurrences ≥3 are enriched in genes belonging to a protein complex, versus only 0.7% of randomly generated clusters with the same sizes. The enrichment fold ratio for protein complexes increases with the cluster recurrence (Figure 2D). The five most frequently enriched protein complexes are "Spliceosome", "CCT micro-complex", "large *Drosha *complex", *"Nop56p-*associated pre-rRNA complex", and "C complex spliceosome". At least 1/3 of subunits in the enriched complex "large *Drosha *complex" contain proteins associated with splicing function, especially heterogeneous nuclear ribonucleoproteins such as *HNRNPH1*, *HNRNPM*, *HNRNPU*, *HNRNPUL1 *and *HNRNPDL *[32].

### Co-splicing clusters reveal novel functions that are not identified by co-expression clusters

Studies have shown that genes that are co-regulated transcriptionally do not necessarily overlap with those that are co-spliced [33]. Therefore, the identification of co-splicing clusters can reveal functionally related genes that could not be discovered from transcription analysis. In order to identify novel functions associated with co-splicing but not co-expression, we complement the above analysis by constructing a gene co-expression network from each RNA-seq dataset. The nodes of these networks represent genes, and the edges are weighted by Pearson's correlation between two gene expression profiles. We then apply our tensor-based pattern mining algorithm to identify frequent co-expression clusters in the 38 co-expression networks with the same criteria as those of identifying co-splicing clusters. The same functional enrichment analysis described above for co-splicing clusters was performed on the resulting co-expression clusters. We found that 98.8% of co-splicing clusters with recurrences ≥ 3 have low expression correlations (average correlations ≤ 0.2). Therefore, many of the functions associated with post-transcriptional regulation are enriched in co-splicing clusters but not in co-expression clusters. These functions include "maintenance of protein location", "regulation of protein catabolic process", "cytoplasmic sequestering of protein", "regulation of intracellular protein transport", "regulation of ubiquitin-protein ligase activity", "ribonucleoprotein complex assembly", "RNA splicing, via transesterification reactions", and "RNA export from nucleus".

For example, one co-splicing cluster has seven host genes: *HNRNPUL1*, *HNRNPC*, *DHX9*, *BAT1*, *PSMA5*, *RAD23 *and *RPS9*. This cluster cannot be found from co-expression data, for the expression profiles of the host genes have low correlations. However, this set of host genes is enriched with several splicing associated functions, including "RNA splicing" (*p*-value = 1.89E-6) and "RNA helicase activity" (*p*-value = 4.68E-5). Out of seven host genes, *HNRNPUL1 *and *HNRNPC *belong to heterogeneous nuclear ribonucleoprotein family, which generally suppress splicing through binding to exonic splicing silencer [2]. *DHX9*, known as *RNA helicase A*, is a highly conserved DEAD-box protein that activates transcription, modulates RNA splicing, binds the nuclear pore complex and involves in spliceosome assembly [34,35]. Previous research illustrated that *DHX9 *mediates association of *CBP *and *RNA polymerase II *[36], and current study further shows that *DHX9 *interacts with post-transcriptional control element RNA in the nucleus and cytoplasm to facilitate efficient translation [34]. Interestingly, *HNRNPC *and *DHX9 *are indeed tightly functionally associated: silencing of *DHX9 *seriously disturbed the nuclear distribution of the *hnRNP C *protein [37]. As an essential splicing factor, *BAT1 *also belongs to DEAD-box protein family, and plays an important role in mRNA export from the nucleus to the cytoplasm, supported by recent experimental evidence that knocked down BAT1 induces spliced mRNA, as well as total polyA RNA accumulating in nuclear speckle domains, not exporting to the cytoplasm [38]. Clearly, co-splicing clusters can provide complementary information on functionally related gene groups in addition to co-trancriptional clusters. In particular, co-splicing clusters can grant new insights into functions associated with post-transcriptional regulation.

### Exons can dynamically participate in different pathways upon different co-splicing mechanisms

Alternatively skipping or including a cassette exon can change the functions of a protein by deleting or inserting a protein domain. In other words, protein isoforms alternatively spliced from the same gene may participate in different pathways. In our results, we observed that 70.3%/52.3%/38.3%/27.1% of exons are members of at least two clusters (recurrence≥3/4/5/6) with different functions. For example, exon8 of the gene *Rela *appears in four co-splicing clusters with recurrences ≥3, which are enriched with the following distinct functions respectively: "ER-associated protein catabolic process" (*p*-value = 2.20E-5), "response to extracellular stimulus" (*p*-value = 3.80E-5), "regulation of gene-specific transcription" (*p*-value = 8.89E-5), and "positive regulation of intracellular protein kinase cascade" (*p*-value = 2.49E-5). *Rela *encodes the transcription factor *p65*, which is an important subunit of the *NF-κB *complex that affects several hundred genes by *NF-κB *signaling. Recent research has identified several alternative splice variants of *Rela*, e.g. *p65A*, *p65A2 *and *p65A3*. In fact, *p65A *arises by the use of an alternative splice site located 30 nucleotides into exon8, and *p65A3 *was identified as a splice variant lacking exon7 and exon8 [39]. These facts are consistent with our finding that exon8 is dynamically included in multiple co-splicing clusters. As another example, exon2 of the gene *EIF5 *appears in three co-splicing clusters with recurrences ≥3, which are enriched with following distinct functions respectively: "RNA splicing" (*p*-value = 6.27E-5), "mRNA polyadenylation" (*p*-value = 1.57E-5), and "regulation of translational initiation" (*p*-value = 8.18E-5). As a translation initiation factor, *EIF5 *plays critical roles for the accurate recognition of correct start codon during translation initiation [40]. Our result suggests that except for translation initiation regulation, *EIF5 *may also involve in post-transcriptional regulation, such as RNA splicing and mRNA polyadenylation by dynamically including exon2 in multiple co-splicing clusters. These examples demonstrate that exons can contribute to different functionalities of proteins depending on different splicing regulatory mechanisms.

## Conclusions

Splicing code is determined by a combination of many factors, such as cis-regulatory elements and transacting factors. If some exons share the same splicing code, they may form a splicing module: a unit in the splicing regulatory network. Therefore, identifying co-splicing clusters first and then investigating their cis-regulatory elements and associated trans-acting factors can serve as an important step to decipher the splicing code. Our tensor-based approach can identify co-spliced exon clusters that frequently appear in multiple RNA-seq datasets. The exons in a frequent co-splicing cluster can belong to different genes, but are very likely to be co-regulated by the same splicing factors, thus forming a splicing module. We demonstrated that the identified clusters represent meaningful biological modules, i.e. functional modules, splicing modules, transcriptional modules, and protein complexes, by validating against four biological knowledge databases. In all four types of enrichment results, the likelihood that a co-splicing cluster is biologically meaningful increases with its recurrence. This consistent behavior highlights the importance of the integrative approach. We also showed that the co-splicing clusters can reveal novel functional related genes that cannot be identified by co-expression clusters, and that the same exons can dynamically participate in different pathways depending on different conditions and different other exons that are co-spliced. The *NCBI Sequence Read Archive *database currently stores 8099 RNA-seq profiles, and this number is expected to dramatically increase in the near future. We expect to apply our approach to the rapidly accumulating RNA-seq data of multiple organisms, and to identify a large number of splicing modules and their associated phenotype conditions. This analysis can serve as a first step towards the reconstruction of tissue- and disease-specific splicing regulatory networks.

## Methods

Given an RNA-seq dataset, we construct a co-splicing network where nodes represent exons and edges are weighted by the correlation between two exon inclusion rate profiles. Given *m *co-splicing networks with the same *n *nodes but different edge weights, we can represent the whole system as a 3^rd^-order tensor $A={\left(a_{ijk}\right)}_{n\times n\times m}$. A *frequent co-splicing cluster *(FSC) in the tensor  can be defined by two membership vectors: (i) the *exon membership vector ***x **= (*x*_1_, ..., *x*_*n*_)^*T*^, where *x*_*i *_= 1 if exon *i *belongs to the cluster and *x*_*i *_= 0 otherwise; and (ii) the *network membership vector ***y **= (*y*_1_, ..., *y*_*m*_)^*T*^, where *y*_*j *_= 1 if the exons of the cluster are heavily interconnected in network *j *and *y*_*j *_= 0 otherwise. The summed weight of all edges in the FSC is

(1) $$H_{A}\left(x,y\right)=\frac{1}{2}\overset{n}{\underset{i=1}{\sum }}\overset{n}{\underset{j=1}{\sum }}\overset{m}{\underset{k=1}{\sum }}a_{ijk}x_{i}x_{j}y_{k}.$$

Note that only the weights of edges *a*_*ijk *_with *x*_*i *_= *x*_*j *_= *y*_*k *_= 1 are counted in $H_{A}$. Thus, $H_{A}\left(x,y\right)$ measures the "heaviness" of the FSC defined by **x **and **y**. The problem of discovering a frequent co-splicing cluster can be formulated as a discrete combinatorial optimization problem: *among all patterns of fixed size (K*_1 _*member exons and K*_2 _*member networks), we look for the heaviest*. This is also an integer programming problem: find the binary membership vectors **x **and **y **that jointly maximize $H_{A}$ under the constraints $\sum_{i=1}^{n}x_{i}=K_{1}$ and $\sum_{j=1}^{m}y_{j}=K_{2}$. However, there are several major drawbacks to this discrete formulation.

The first is *parameter dependence, *meaning that the size parameters *K*_1 _and *K*_2 _are hard for users to provide and control. The second is *high computational complexity*; the task is proved to be NP-hard (see additional file 1: Section S1) and therefore not solvable in a reasonable time even for small datasets. Therefore, the discrete optimization problem is infeasible for an integrative analysis of many massive networks. Instead, we solve a continuous optimization problem with the same objective by relaxing integer constraints to continuous constraints. That is, we look for non-negative real vectors **x **and **y **that jointly maximize $H_{A}$. This optimization problem is formally expressed as follows:

(2) $$\begin{array}{c} {\text{max}}_{x\in {\mathbb{R}}_{+}^{n},y\in {\mathbb{R}}_{+}^{m}}\;H_{A}\left(x,y\right)\\ \text{subject}\;\text{to}\;f\left(x\right)=1\;\;and\;\;g\left(y\right)=1 \end{array}$$

where ℝ_+ _is a non-negative real space, and *f*(**x**) and *g*(**y**) are vector norms. After solving Eq. (2), users can easily identify the top-ranking networks (after sorting the tensor by **y**) and top-ranking exons (after sorting each network by **x**) contributing to the objective function. After rearranging the networks in this manner, the FSC with the largest heaviness occupies a corner of the 3D tensor. We can then mask all edges in the heaviest FSC with zeros, and optimize Eq. (2) again to search for the next FSC.

The choice of vector norms in Eq. (2) has a significant impact on the outcome of the optimization. A vector norm defined as ${∥x∥}_{p}={\left(\sum_{i=1}^{n}{\left|x_{i}\right|}^{p}\right)}^{1/p}$, where *p *> 0, is also called an "*L*_*p*_-vector norm". In general, the closer *p *is to zero, the sparser the solution favored by the *L*_*p*_-norm; that is, fewer components of the optimized vectors are significantly different from zero [41]. In contrast, as *p *increases, the solution favored by the *L*_*p*_-norm grows smoother; in the extreme case *p *→ ∞, the elements of the optimized vector are approximately equal to each other. For more details on these vector norms, refer to the additional file 1: Section S2. Our ideal membership vector selects *a small number of exons ("sparse") whose values are close to each other in magnitude ("smooth"), while the rest of exons are close to zero. *Our past research [12] has shown that this goal can be achieved using the mixed norm *L*_0,__∞_(**x**) = *α*∥**x**∥_0 _+ (1 - *α*)∥**x**∥_∞ _(0 <*α *< 1) for *f*(**x**). The norm *L*_0 _favors sparsity while the norm *L*_∞ _encourages smoothness in the non-zero components of **x**. In practice, we approximate *L*_0,__∞_(**x**) with another mixed norm: *L*_*p*,2_(**x**) = *α*∥**x**∥_*p *_+ (1 - *α*)∥**x**∥_2_, where *p *< 1. Our criteria for the network membership vector are similar. We want the exon cluster to appear in as many networks as possible, so the network membership values should be non-zero and close to each other. This is the typical outcome of optimization using the *L*_∞ _norm. In practice, we approximate *L*_∞ _with *L*_*q*_(**y**), where *q *> 1 for *g*(**y**). Therefore, the vector norms *f*(**x**) and *g*(**y**) are fully specified as follows,

(3) $$f\left(x\right)=\alpha {∥x∥}_{p}+\left(1-\alpha \right){∥x∥}_{2}\;\;\text{and}\;\;g\left(y\right)={∥y∥}_{q}$$

We performed simulations to determine suitable values for the parameters *p*, *α*, and *q*, by applying our tensor method to collections of random weighted networks. We randomly placed FSCs of varying size, recurrence, and heaviness in a subset of the random networks. We then tried different combinations of *p*, *α*, and *q*, and adopted the combination (*p *= 0.8, *α *= 0.2, and *q *= 10) that led to the discovery of the most FSCs. More details on these simulations are provided in the additional file 1 (Section S4).

Since the vector norm *f*(**x**) is non-convex, our tensor method requires an optimization protocol that can deal with non-convex constraints. The quality of the optimum discovered for a non-convex problem depends heavily on the numerical procedure. Standard numerical techniques such as gradient descent converge to a local minimum of the solution space, and different procedures often find different local minima. Thus, it is important to find a theoretically justified numerical procedure. We use an advanced framework known as multi-stage convex relaxation, which has good numerical properties for non-convex optimization problems [41]. In this framework, concave duality is used to construct a sequence of convex relaxations that give increasingly accurate approximations to the original non-convex problem. We approximate the sparse constraint function *f*(**x**) by the convex function ${\tilde{f}}_{v}\left(x\right)=v^{T}h\left(x\right)-f_{h}^{*}\left(v\right)$, where *h*(**x**) is a specific convex function *h*(*x*) = *x*^2 ^and $f_{\hbar }^{*}\left(v\right)$ is the concave dual of the function ${\bar{f}}_{h}\left(v\right)$ (defined as $f\left(v\right)={\bar{f}}_{h}\left(h\left(v\right)\right)$). The vector **v **contains coefficients that will be automatically generated during the optimization process. After each optimization, the new coefficient vector **v **yields a convex function ${\tilde{f}}_{v}\left(x\right)$ that more closely approximates the original non-convex function *f*(**x**). Details of our tensor-based optimization method can be found in the additional file 1 (Section S3).

Once the membership vectors (i.e., the solution of Eq. (2)) have been found by optimization, the frequent co-splicing clusters can be intuitively obtained by including those exons and networks with large membership values. However, any given solution can result in multiple overlapping patterns whose "heaviness" is greater than a specified threshold. Here, *heaviness *is defined as the average weight of all edges in the pattern. To identify the most representative pattern, we first rank exons and networks in decreasing order of their membership values in $\hat{x}$ and $\hat{y}$. Then we extract two representative patterns that satisfy the heaviness threshold: the pattern that occurs in the most networks while having at least the minimum number of top-ranking exons (e.g., 5), and the pattern with the largest number of top-ranking exons while appearing in at least the minimum number of top-ranking networks (e.g., 3). Both patterns are included as co-splicing clusters in our results. After discovering a pattern, we can mask its edges in those networks where they occur (replacing those elements of the tensor with zeroes) and optimize Eq. (2) again to search for the next frequent co-splicing cluster.

## Competing interests

The authors declare that they have no competing interests.

## Authors' contributions

XJZ conceived the project; DC and WL performed the research; DC, WL, and XJZ wrote the paper; JL provided input and suggestions. All authors read and approved the final manuscript.

## Supplementary Material

Additional file 1 **Supplementary material**. Additional file provides supplementary material which gives details of data processing and methods. Click here for file
