# Supplementary material for: Integrating many co-splicing networks to reconstruct splicing regulatory modules
Source: BMC Syst Biol. 2012 Jul 16;6(Suppl 1):S17. doi: 10.1186/1752-0509-6-S1-S17 (PMC3403501; doi:10.1186/1752-0509-6-S1-S17)
Supplement: Additional file 1 — Supplementary material. Additional file provides supplementary material which gives details of data processing and methods. [file 1752-0509-6-S1-S17-S1.pdf]

Supplementary Material

Integrating Many Co-Splicing Networks to Reconstruct Splicing Regulatory Modules

Chao Dai *
School of Computer, Wuhan University, Wuhan 430072, PR China

Molecular and Computational Biology, University of Southern California
Los Angeles, CA 90089, USA

Wenyuan Li *
Molecular and Computational Biology, University of Southern California
Los Angeles, CA 90089, USA

Juan Liu
School of Computer, Wuhan University, Wuhan 430072, PR China

Xianghong Jasmine Zhou †
Molecular and Computational Biology, University of Southern California
Los Angeles, CA 90089, USA
xjzhou@usc.edu

*Equally contributed joint first authors.

†To whom correspondence should be addressed.

Contents

S1 NP-hardness of the Heaviest (K_1, K_2)-Frequent Heavy Subgraph Problem **S3**

S2 Details of vector norms **S4**

S3 Tensor-Based Optimization Method **S4**

S3.1 Concave duality S5

S3.2 Multi-stage convex relaxation S5

S4 Simulation study **S7**

S5 RNA-Seq Datasets Selection and Processing, and Network Construction **S7**

S5.1 Cassette Exons Identification S7

S5.2 RNA-Seq Datasets Selection and Processing S8

S5.3 Network Construction S8

S5.4 Non-Uniform Sampling for Fast Computation S9

S6 Descriptions of 38 RNA-Seq Datasets **S11**

S7 Signal extraction procedure of the Encyclopedia of DNA Elements (ENCODE) data **S14**

S1 NP-hardness of the Heaviest (K_1, K_2)-Frequent Heavy Subgraph Problem

The frequent co-splicing cluster can be also called as a graph terminology "frequent heavy subgraph". In this supplementary material, we will use the term "frequent heavy subgraph", not "frequent co-splicing cluster".

Given a set of m undirected graphs $G_1, \ldots, G_m$ with the same n vertices V but different topologies (and without self-loops), i.e., $\mathcal{G} = \{G_1(V, E_1), \ldots, G_m(V, E_m)\}$ with $V = \{v_1, \ldots, v_n\}$ and non-negative weights a_{ijk} for edges $(v_i, v_j) \in E_k$ in the kth graph, the (K_1, K_2)-FHS problem is formally defined as follows,

Problem S1.1. *Given $\mathcal{G}$, the (K_1, K_2)-*Frequent Heavy Subgraph (FHS) *problem is to determine a subset $S_V \in V$ of K_1 vertices and a subset $S_G \in \mathcal{G}$ of K_2 graphs such that the total sum of edge weights of the subgraphs induced by S_V in each graph of S_G is maximized. A straightforward cubic 0-1 formulation of (K_1, K_2)-FHS is*

$$\begin{array}{ll} \max & \frac{1}{2}\sum_{i=1}^{n}\sum_{j=1}^{n}\sum_{k=1}^{m} a_{ijk}x_i x_j y_k \\ \textit{subject to} & \begin{cases} \sum_{i=1}^{n} x_i = K_1 & \\ \sum_{i=1}^{m} y_j = K_2 & \\ x_i \in \{0,1\} & \textit{for any } 1 \leqslant i \leqslant n \\ y_j \in \{0,1\} & \textit{for any } 1 \leqslant j \leqslant m \end{cases} \end{array} \tag{1}$$

Next we prove the NP-hardness of this problem.

Theorem S1.2. *The (K_1, K_2)-frequent heavy subgraph problem is NP-hard.*

Proof. We can reduce the well-known NP-complete K-clique problem [1] (i.e., *is there a clique with K vertices in a graph?*) to this problem, and therefore prove its NP-hardness.

Let $G(V, E)$ be an undirected unweighted graph without self-loops. We can copy this graph m times to generate a graph set consisting of the m graphs $\mathcal{G} = \{G_1(V, E), \ldots, G_m(V, E)\}$ in which all graphs have the same vertex set V and edge set E. Then the question of "*is there a clique with K vertices in G?*" can be answered by solving the (K, K_2)-FHS problem in the set of m graphs $\mathcal{G}$, because we can easily claim:

- *If the heaviest (K, K_2)-FHS found in the graph set $\mathcal{G}$ is a clique with K vertices recurring in the K_2 graphs, then there exist a clique with K vertices in G.* This claim is obvious and straightforward.
- *If the heaviest (K, K_2)-FHS found in the graph set $\mathcal{G}$ is not a clique with K vertices recurring in the K_2 graphs, then a clique with K vertices does not exist in G.* This claim can be proved by contradiction: supposing there exists a clique with K vertices in G, since all graphs G_i in the graph set $\mathcal{G}$ were copied from G, this K-clique in G must also exist at least K_2 graphs of the graph set $\mathcal{G}$. Among all (K, K_2)-FHSs in m unweighted graphs, the K-cliques recurring in K_2 graphs must be the (K, K_2)-FHS having the largest total sum of edge weights. This K-clique recurring in K_2 graphs must be the solution of the heaviest (K, K_2)-FHS. So it contradicts with the statement "the heaviest K, K_2-FHS found is not a clique with K vertices recurring in the K_2 graphs".

[1] The NP-completeness proof of the K-clique problem can be found in textbooks introducing "algorithms and complexity" and lectures' exercises, e.g., `http://people.bath.ac.uk/masnnv/Teaching/AAlg10Sol.pdf`.

Since the K-clique problem is NP-complete, the *(K_1, K_2)-FHS* problem is NP-hard. □

S2 Details of vector norms

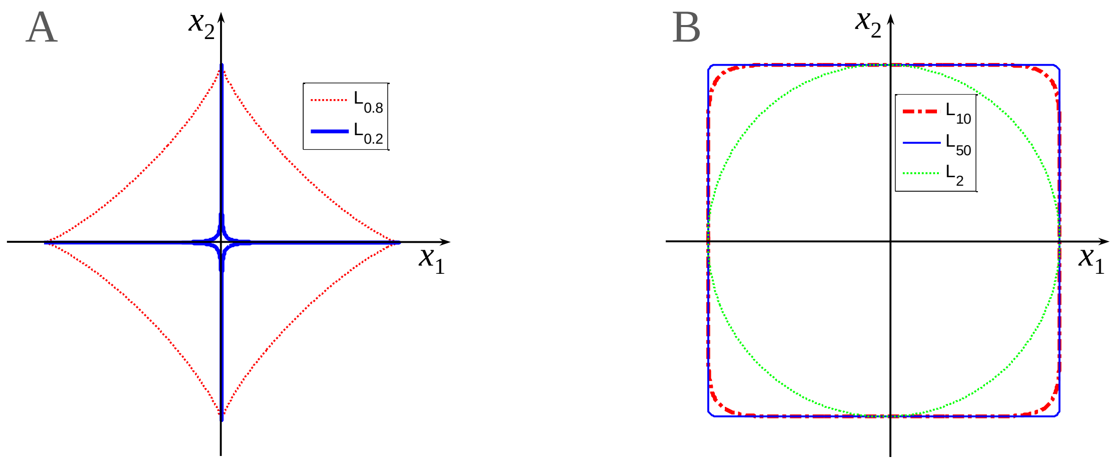

Figure S1. Two-dimensional contour plots of the vector norm constraints. (A) contour of the vector norm L_p $(0 < p < 1)$. When $p \to 0$, $L_p \to L_0$ and therefore L_p can make $\mathbf{x} = (x_1, x_2)^T$ sparser. (B) Contour of the vector norm L_p $(p > 1)$. When $p \to \infty$, $L_p \to L_\infty$ and therefore L_p can make $\mathbf{x} = (x_1, x_2)^T$ more even

The L_p $(p > 0)$ norm of a vector $\mathbf{x} \in \mathbb{R}^{n\times 1}$ is defined as $\|\mathbf{x}\|_p = (\sum_{i=1}^{n} |x_i|^p)^{1/p}$. Two extreme cases of L_p norm is zero norm $L_0 = \text{card}\{x_i | x_i \neq 0\}$, where card is the set cardinality (i.e., the number of non-zero elements of $\mathbf{x}$), and infinity norm (or maximum norm) $L_\infty = \max\{x_1, \ldots, x_n\}$. When the L_p vector norm is used as optimization's constraint, the set of all vectors with norm 1 (i.e., $L_p(\mathbf{x}) = 1$) defines the feasible region, whose two-dimensional case is shown in Figure S1. It can be observed that the closer p is to zero, the sparser $\mathbf{x}$ is; while the closer p is to ∞, the smoother or more even $\mathbf{x}$ is. In practice, L_p with $p < 1$ is often used to approximate L_0, and L_p with $p \geqslant 2$ is often used to approximate L_∞.

S3 Tensor-Based Optimization Method

Since the vector norm $f(\mathbf{x})$ is non-convex, our tensor method requires an optimization protocol that can deal with non-convex constraints. While the global optimum of a convex problem can be easily computed, the quality of the optimum discovered for a non-convex problem depends heavily on the numerical procedure. Standard numerical techniques such as gradient descent converge to a local minimum of the solution space, and different procedures often find different local minima. Considering the fact that our sparse constraint is non-convex, it is important to find a theoretically justified numerical procedure. We use an advanced optimization framework known as multi-stage convex relaxation, which has good numerical properties for non-convex optimization problems [1]. In this context, concave duality is used to construct a sequence of convex relaxations that give increasingly accurate approximations to the original non-convex problem. We approximate the sparse constraint function $f(\mathbf{x})$ by the convex function $\widetilde{f}_{\mathbf{v}}(\mathbf{x}) = \mathbf{v}^T h(\mathbf{x}) - f_h^*(\mathbf{v})$, where $h(\mathbf{x})$ is

a specific convex function $h(x) = x^h$ ($h \geqslant 1$) and $f_h^*(\mathbf{v})$ is the concave dual of the function $\overline{f}_h(\mathbf{v})$ (defined as $f(\mathbf{v}) = \overline{f}_h(h(\mathbf{v}))$). In practice, $h = 2$ is an effective choice as the convex upperbound of $f(\mathbf{x})$. The vector $\mathbf{v}$ contains coefficients that will be automatically generated during the optimization process. After each optimization, the new coefficient vector $\mathbf{v}$ yields a convex function $\widetilde{f}_{\mathbf{v}}(\mathbf{x})$ that more closely approximates the original non-convex function $f(\mathbf{x})$.

S3.1 Concave duality

Given a continuous regularization function $f(\mathbf{x})$ which may be non-convex, we are interested in rewriting it using concave duality. Detail refer to [1]. Let $\mathbf{h}(\mathbf{x}) : \mathbb{R}^n \to \Omega \subset \mathbb{R}^n$ be a vector function. It may not be a one-to-one map. However, we assume that there exists a function $\overline{f}_{\mathbf{h}}(\mathbf{u})$ defined on Ω such that $f(\mathbf{x}) = \overline{f}_{\mathbf{h}}(\mathbf{h}(\mathbf{x}))$ holds.

We assume that we can find $\mathbf{h}$ so that the function $\overline{f}_{\mathbf{h}}(\mathbf{u})$ is a concave function of $\mathbf{u}$ on Ω. Under this assumption, we can rewrite the regularization function $f(\mathbf{x})$ as:

$$f(\mathbf{x}) = \inf_{\mathbf{v}\in\mathbb{R}^n} \left[\mathbf{v}^T \mathbf{h}(\mathbf{x}) - f_{\mathbf{h}}^*(\mathbf{v})\right] \tag{2}$$

using concave duality (Page 308 in [2]). In this case, the function $f_{\mathbf{h}}^*(\mathbf{v})$ given below is the *concave dual* of $\overline{f}_{\mathbf{h}}(\mathbf{u})$:

$$f_{\mathbf{h}}^*(\mathbf{v}) = \inf_{\mathbf{u}\in\Omega} \left[\mathbf{v}^T \mathbf{u} - \overline{f}_{\mathbf{h}}(\mathbf{u})\right] \tag{3}$$

Moreover, it is well-known that the minimum of the right hand side of Eq. (2) is achieved at

$$\hat{\mathbf{v}} = \nabla_{\mathbf{u}} \overline{f}_{\mathbf{h}}(\mathbf{u})|_{\mathbf{u}=\mathbf{h}(\mathbf{x})} \tag{4}$$

This is a general framework. As $f(\mathbf{x}) = \alpha||\mathbf{x}||_p + (1-\alpha)||\mathbf{x}||_2$ for some $p \in (0, 1)$, given any $h \geqslant 2$, Eq. (2) holds with $\mathbf{h}(\mathbf{x}) = \left[|x_1|^h, \ldots, |x_n|^h\right]$. The solution in (4) is given by,

$$\hat{v}_i = \frac{\alpha}{h}(\sum_j |x_j|^p)^{\frac{1}{p}-1}|x_i|^{p-h} + \frac{1-\alpha}{h}(\sum_j x_j^2)^{\frac{1}{2}-1}|x_i|^{2-h} \tag{5}$$

S3.2 Multi-stage convex relaxation

The solution of our tensor formulation is a stationary point of the following regularized optimization problem:

$$[\hat{\mathbf{x}}, \hat{\mathbf{y}}] = \arg \max_{\mathbf{x}\in\mathbb{R}^n, \mathbf{y}\in\mathbb{R}^m} \left[\frac{1}{2}\sum_{i,j,k} a_{ijk}x_i x_j y_k - \lambda f(\mathbf{x}) - \mu g(\mathbf{y})\right] \tag{6}$$

where $\lambda > 0$ and $\mu > 0$ are Lagrange multipliers. Since $f(\mathbf{x})$ is non-convex and $g(\mathbf{y})$ is convex, we consider a numerical procedure for solving Eq. (6) with convex loss and non-convex regularization $f(\mathbf{x})$. Let $h(\mathbf{x}) = \sum_j \mathbf{h}_j(\mathbf{x})$ be a convex relaxation of $f(\mathbf{x})$ that dominates $f(\mathbf{x})$ (for example, the smallest convex upperbound, i.e., the inf over all convex upperbounds). A simple convex relaxation of Eq. (6) becomes

Inputs: tensor $\mathcal{A} = (a_{ijk})_{n\times n\times m}$, initial values $\mathbf{x}^{(0)} \in \mathbb{R}^n$ and $\mathbf{y}^{(0)} \in \mathbb{R}^m$.
Outputs: the exon membership vector $\mathbf{x}$ and network membership vector $\mathbf{y}$
Initialize $\hat{v}_j = 1$.
Repeat the following two steps (a stage) until convergence:

- Step 1: let $[\widehat{\mathbf{x}}, \widehat{\mathbf{y}}] = \arg\max_{\mathbf{x}\in\mathbb{R}_+^n, \mathbf{y}\in\mathbb{R}_+^m} \left[\frac{1}{2}\sum a_{ijk}x_ix_jy_k - \lambda\hat{\mathbf{v}}^T h(\mathbf{x}) - \mu g(\mathbf{y})\right]$.
- Step 2: let $\widehat{\mathbf{v}} = \nabla_{\mathbf{u}}\overline{f}_h(\mathbf{u})|_{\mathbf{u}=h(\mathbf{x})}$.

Figure S2. Multi-stage convex relaxation method for the tensor-based problem.

$$[\hat{\mathbf{x}}, \hat{\mathbf{y}}] = \arg \max_{\mathbf{x}\in\mathbb{R}^n, \mathbf{y}\in\mathbb{R}^m} \left[\frac{1}{2}\sum_{i,j,k} a_{ijk}x_ix_jy_k - \lambda\sum_{j=1}^{n}\mathbf{h}_j(\mathbf{x}) - \mu g(\mathbf{y})\right] \tag{7}$$

It is possible that this simple relaxation yields a solution that is not close to the solution of (6). However, if $\mathbf{h}$ satisfies the condition of Subsection S3.1, then it is possible to write $f(\mathbf{x})$ as Eq. (2). In this new representation, we can rewrite Eq. (6) as

$$[\hat{\mathbf{x}}, \hat{\mathbf{y}}, \hat{\mathbf{v}}] = \arg\max_{\mathbf{x},\mathbf{v},\mathbf{y}} \left[\frac{1}{2}\sum_{i,j,k} a_{ijk}x_ix_jy_k - \lambda\mathbf{v}^T\mathbf{h}(\mathbf{x}) + \lambda f_{\mathbf{h}}^*(\mathbf{v}) - \mu g(\mathbf{y})\right] \tag{8}$$

This is clearly equivalent to Eq. (6) because of Eq. (2). If we can find a good approximation of $\hat{\mathbf{v}}$ that improves upon the initial value of $\hat{\mathbf{v}} = [1, \ldots, 1]^T$, then the above formulation can lead to a refined problem in $\mathbf{x}$ that is a better relaxation than Eq. (7).

Our numerical procedure exploits the above fact, trying to improve the estimation of v_j over the initial choice of $v_j = 1$ in Eq. (8) using an iterative algorithm. This can be done by repeatedly applying the following two steps:

- First, optimize $\mathbf{x}$ and $\mathbf{y}$ with $\mathbf{v}$ fixed.
- Second, optimize $\mathbf{v}$ with $\mathbf{x}$ and $\mathbf{y}$ fixed. This problem has the closed form solution given by Eq. (4).

Figure S2 presents our two-stage protocol to solve the regularized form of our problem. The procedure can be regarded as a generalization of concave-convex programming [3], which takes $h(\mathbf{x}) = \mathbf{x}$. By repeatedly refining the parameters in $\mathbf{v}$, we can obtain better and better convex relaxations leading to a solution superior to that of the initial convex relaxation with $v_j = 1$. The initial values of $\mathbf{x}$ and $\mathbf{y}$ could be uniform, randomly chosen, or taken from prior knowledge. In practice, an appropriate solver for Step 1, the time complexity of MSCR is often linear with respect to the total number of edges in the tensor.

Let $\mathbf{h}(\mathbf{x}) = \left[|x_1|^h, \ldots, |x_n|^h\right]$ and $h = 2$. This function is practically effective as the small convex upperbound of $f(\mathbf{x})$. The problem in Step 1 of Figure S2 can be approximately implemented by the power method presented in Figure S3. However instead of tuning λ and μ, this is implicitly done by using the gradient projection scheme so that the constraints $f(\mathbf{x}) = 1$ and $g(\mathbf{y}) = 1$ are enforced

after each iteration via normalization. The idea is to optimize $\mathbf{x}$ with $\mathbf{y}$ fixed, then normalize $\mathbf{x}$ such that $\mathbf{x}$ satisfies the constraint $f(\mathbf{x}) = 1$. Similarly, we optimize $\mathbf{y}$ with $\mathbf{x}$ fixed, then normalize $\mathbf{y}$ such that $\mathbf{y}$ satisfies the constraint $g(\mathbf{y}) = 1$. The initial values $\mathbf{x}^{(0)}$ and $\mathbf{y}^{(0)}$ can be the vector with all entries one, i.e., $\mathbf{1} = [1, \ldots, 1]^T$. As discussed in Section S3.1, the solution of Step 2 is given in Eq. (5) when $f(\mathbf{x})$ is relaxed to $\mathbf{h}(\mathbf{x})$.

Inputs: tensor $A = (a_{ijk})_{n\times n\times m}$, vector $\hat{\mathbf{v}} \in \mathbb{R}^n$, initial values $\mathbf{x}^{(0)} \in \mathbb{R}^n$ and $\mathbf{y}^{(0)} \in \mathbb{R}^m$
Outputs: $\hat{\mathbf{x}}$ and $\hat{\mathbf{y}}$
Initialize $\mathbf{x} = \mathbf{x}^{(0)}$, $\mathbf{y} = \mathbf{y}^{(0)}$.
Repeat the following two updates until convergence:

- Update $\mathbf{x}$: $x_i \leftarrow \left[\frac{x_i \sum_{j,k} a_{ijk} x_j y_k}{\hat{v}_i}\right]^{\frac{1}{h}}$, then $\mathbf{x}$ is normalized by $x_i \leftarrow \frac{x_i}{\alpha||\mathbf{x}||_p + (1-\alpha)||\mathbf{x}||_2}$.
- Update $\mathbf{y}$: $y_k \leftarrow (y_k \sum_{i,j} a_{ijk} x_i x_j)^{\frac{1}{q}}$, then $\mathbf{y}$ is normalized by $y_k \leftarrow \frac{y_k}{||\mathbf{y}||_q}$.

Figure S3. Power method for updating $\mathbf{x}$ and $\mathbf{y}$ in Step 1 of the MSCR method shown in Figure S2

S4 Simulation study

We generated 100 sets of random weighted networks with 300 exons, where each set contains 50 networks and 300 exons, and all their edges weights follow the uniform distribution in the range $[0, 1]$. Then a random Frequent Heavy Subgraph (FHS) patterns with K_1 member exons and K_2 member networks are generated by making their edges weights follow the uniform distribution in the range $[\theta, 1]$. Here, K_1 is any of the four predefined values $\{6, 12, 18, 24\}$, K_2 is any of the five predefined values $\{5, 10, 15, 20, 25\}$, and θ is any of the five predefined values $\{0.5, 0.6, 0.7, 0.8.0.9\}$. Therefore, there are the total $4 \times 5 \times 5 = 100$ FHS patterns generated. Each FHS pattern is then placed into a set of networks, by randomly selecting K_1/K_2 exons/networks and replacing edges weights with the corresponding FHS pattern's edges weights. These simulated networks and patterns have taken into account of various factors that may affect the performance. We can evaluate the performance by counting the number of these predefined patterns found/hitted by the method with each parameter combination.

We performed our tensor method with different values of the parameters p and α on each set of networks and obtained the result as shown in Table S1. As explained in this table, $p = 0.8$ and $\alpha = 0.2$ is one of the best choices.

S5 RNA-Seq Datasets Selection and Processing, and Network Construction

S5.1 Cassette Exons Identification

We compiled human cassette exons from *hg19* UCSC gene annotations downloaded from UCSC [4]. For every gene, firstly we removed exons in 5'UTR and 3' UTR of each transcript, and then checked

p \ α	0.2	0.4	0.6	0.8
0.2	1%	1%	1%	1%
0.4	31%	29%	28%	27%
0.6	100%	100%	100%	100%
0.8	100%	100%	100%	100%

Table S1. Hits table of performing our tensor method with different p and α on all 100 sets of networks, where hit $= \frac{\text{number of predefined patterns obtained/hit by tensor method}}{\text{number of all predefined patterns}}$. In this table, we chose the $\alpha = 0.2$ whose hitting values are always $\geqslant$ those of other α given the same p; similarly, we chose the $p = 0.8$ whose hitting values are always $\geqslant$ those of other p given the same α. They are highlighted by graying the first column and the last row.

the location of each remaining exon to determine whether it is contained in all the known transcripts belonging to the gene model. If an exon is not contained in all the transcripts, the exon is a cassette exon. We applied this method to all the UCSC genes, and identify 22,310 cassette exons in human genome. It is worth noting that some cassette exons are always contained in the same transcripts, which means they are always co-spliced together as long as the transcripts are expressed. Since this trivial case dominates heaviness calculation in co-splicing clusters, and produce biased result, we combined those cassette exons that are always included in the same transcripts as a cluster, and used one cassette exon to represent the whole cassette exon clusters.

S5.2 RNA-Seq Datasets Selection and Processing

From NCBI's Sequence Read Archive (SRA) we selected all human RNA-seq datasets, each of which contains at least six samples (the minimum for robust correlation estimation). This results in a total of 38 datasets. For each dataset, we used the Tophat [5] tool to map short reads to the *hg19* reference genome and applied the transcript assembly tool Cufflinks [6] to estimate expressions for all transcripts with known UCSC transcript annotations [4]. We calculated the inclusion rate of each exon in every sample, as the ratio between its expression (i.e., sum of FPKM over all transcripts that cover the exon) and the expression of the host gene (i.e., sum of FPKM over all transcripts of the gene). It is worth noting that in RNA-seq experiments, a gene expression with low FPKM is usually not precisely estimated because the number of reads mapped to the gene is quite small. In order to work with reasonably accurate estimates of exon inclusion rates, as pointed out by [7], we calculated inclusion rates only for those exons whose host genes' expressions are above 80th percentile across at least 6 samples. Throughout this study, we only considered the genes containing cassette exons whose inclusion rate profiles met the above criterion. This resulted in 16,024 exons covering 9,532 genes. The 38 datasets that met these criteria on January 30 2011 were used for the analysis described herein.

S5.3 Network Construction

For each RNA-seq dataset containing a set of samples, a *weighted exon co-splicing network* can be constructed where the nodes represent exons and the edge weights are correlations between the inclusion rates of two exons. To determine the weights, we first compute the correlation between two exons as the leave-one-out Pearson correlation coefficient estimate [8]. The resulting

correlation estimate is conservative and sensitive to similarities in the patterns, yet robust to single experimental outliers. To make the correlation estimates comparable across datasets, we then applied Fisher's z transform [9]. Given a correlation estimate r, Fisher's transformation score is calculated as $z = \frac{\sqrt{n-3}}{2} \ln\left(\frac{1+r}{1-r}\right)$. Note that the sample size n may be different for different datasets and even for different exon pairs due to missing values. Practically, we observed the distributions of z-scores may still vary from dataset to dataset, we standardized the z-scores to enforce zero mean and unit variance in each dataset by following the normalization procedure introduced in [10, 11]. Then, the "normalized" correlations r' are obtained by inverting the z-score with the same virtual sample size $n' = 10$: $r' = \frac{\exp(\frac{2}{\sqrt{n'-3}}z)-1}{\exp(\frac{2}{\sqrt{n'-3}}z)+1}$. Finally, the absolute value of r' is used as the edge weight of networks.

S5.4 Non-Uniform Sampling for Fast Computation

Even though our optimization method is efficient, its computation time can still be long for large sets of networks with many edges. In such cases, edge sampling can provide an efficient approximation to many graph problems [12, 13]. From the perspective of matrix or tensor computation, such sampling methods can also be viewed as matrix/tensor sparsification [14]. As FSCs predominately contain edges with large weights, we designed a non-uniform sampling method that preferentially selects edges with large weights. Specifically, given a tensor $\mathcal{A}$, each edge a_{ijk} is sampled with probability p_{ijk}:

$$p_{ijk} = \begin{cases} 1, & \text{if } a_{ijk} \geqslant \tilde{a} \\ p\left(\frac{a_{ijk}}{\tilde{a}}\right)^b, & \text{if } a_{ijk} < \tilde{a} \end{cases} \tag{9}$$

where $\tilde{a} \in (0, 1)$, $b \in [1, \infty)$ and $p \in (0, \tilde{a}^b]$ are constants that control the number of sampled edges. Note that Eq. (9) *always* samples edges with weights $\geqslant \tilde{a}$. It selects an edge of weight $a_{ijk} < \tilde{a}$ with probability p_{ijk} proportional to the bth power of the weight. We choose $\tilde{a} = 0.4$, $b = 3$, and $p = 0.1$ as a reasonable tradeoff between computational efficiency and the quality of the sampled tensor, meanwhile satisfying the conditions of Theorem S5.1, i.e. $p \leqslant \tilde{a}^{b-1}$ and $b \geqslant 1$.

To correct the bias caused by this sampling method, the weight of each edge is corrected by its relative probability: $\widehat{a}_{ijk} = a_{ijk}/p_{ijk}$. The expected weight of the sampled network, $E(\widehat{a}_{ijk})$, is therefore equal to the weight of the original network. However, in practice, when the adjusted edge weight $\widehat{a}_{ijk} > \tilde{a}$ (but the original edge weight $a_{ijk} < \tilde{a}$), we enforced it to be $\widehat{a}_{ijk} = \tilde{a}$ to avoid too large edge weights. The overall edge sampling procedure adopts the simple random-sampling based single-pass sparsification procedure introduced in [14]. Details of the sampling procedure is given below. This single-pass sampling procedure's time complexity is $O(n^2m)$. It is obvious that the sparsification procedure in [14] is a special case of our sampling procedure when all entries of $\mathcal{A}$ are non-negative and $p = 1, \tilde{a} = \frac{\epsilon}{n+n+m}, b = 1$. After edge sampling, the procedure described above will use the corrected tensor $\widehat{\mathcal{A}} = (\widehat{a}_{ijk})_{n\times n\times m}$ instead of the original tensor $\mathcal{A}$.

Edge sampling procedure of the tensor $A = (a_{ijk})_{n\times n\times m}$
Procedure $\textsc{Sampling}\big(\mathcal{A}, p, \tilde{a}, b\big)$
for each $i \in [1,n], j \in [1,n], k \in [1,m]$ **do**
if $a_{ijk} \geqslant \tilde{a}$ **then**
$\widehat{a}_{ijk} = a_{ijk}$
else
$$\widehat{a}_{ijk} = \begin{cases} \min(\frac{a_{ijk}}{p_{ijk}}, \tilde{a}), & \text{with probability } p_{ijk} = p\left(\frac{a_{ijk}}{\tilde{a}}\right)^b \\ 0 & \text{with probability } 1 - p_{ijk} \end{cases}$$
return $\widehat{\mathcal{A}} = (\widehat{a}_{ijk})_{n\times n\times m}$

Based on the well-known Chernoff-Hoeffding bounds [15], we gave the bound of the non-zero entries in the corrected tensor $\widehat{\mathcal{A}}$ after sampling in Theorem S5.1. Therefore, the computational complexity of the tensor MSCR algorithm on the tensor $\widehat{\mathcal{A}}$ after sampling is linear to the number of the non-zero entries of $\widehat{\mathcal{A}}$, i.e., $O(\frac{S}{\tilde{a}})$, with the probability at least $1 - \exp(-\Omega(S'))$, where $S = \sum_{i,j,k} a_{ijk}$ and $S' = \frac{p}{\tilde{a}^b}\sum_{i,j,k} a_{ijk}^b$.

Lemma S5.1. *Given $p \leqslant \tilde{a}^{b-1}$, $b \geqslant 1$ and $0 \leqslant a_{ijk} \leqslant 1$ (for any i, j, k), with probability at least $1 - \exp(-\Omega(S'))$, the tensor $\widehat{\mathcal{A}}$ contains at most $O(\frac{S}{\tilde{a}})$ non-zero entries, where $S = \sum_{i,j,k} a_{ijk}$ and $S' = \frac{p}{\tilde{a}^b}\sum_{i,j,k} a_{ijk}^b$.*

Proof. This proof is similar to Lemma 1 in [14]. Since $S = \sum_{i,j,k} a_{ijk}$, the number of a_{ijk} that are no less than $\tilde{a}$ is at most $\frac{S}{\tilde{a}}$; otherwise, the sum of all entries $\geqslant \tilde{a}$ would be greater than S. Now consider all non-zero a_{ijk} entries that are smaller than $\tilde{a}$.

The Chernoff bound [15] asserts that if $X_1, X_2, \ldots, X_N$ are indicator random variables and $X = \sum_i X_i$ with $\mathbb{E}[X] = \mu$, then for any $\delta > 0$

$$\mathbf{Pr}[X > (1+\delta)\mu] < \left(\frac{e^\delta}{(1+\delta)^{1+\delta}}\right)^\mu \tag{10}$$

In our case, we set up indicator random variables X_{ijk} which are 0 or 1 depending on whether $\tilde{a} = 0$ or not. Then $X = \sum_{i,j,k} X_{ijk}$ is the number of non-zero entries of $\widehat{\mathcal{A}}$, and

$$\mu = \mathbb{E}[X] = \sum_{i,j,k} p_{ijk} = \frac{p}{\tilde{a}^b}\sum_{i,j,k} a_{ijk}^b = S' \tag{11}$$

Since $p \leqslant \tilde{a}^{b-1}$ and $a_{ijk}^b \leqslant a_{ijk}$ (because $b \geqslant 1$ and $0 \leqslant a_{ijk} \leqslant 1$), we have $\frac{p}{\tilde{a}^b}\sum_{i,j,k} a_{ijk}^b \leqslant \frac{1}{\tilde{a}}\sum_{i,j,k} a_{ijk}$. Then, we can get

$$\mu = \frac{p}{\tilde{a}^b}\sum_{i,j,k} a_{ijk}^b \leqslant \frac{1}{\tilde{a}}\sum_{i,j,k} a_{ijk} = \frac{S}{\tilde{a}} \tag{12}$$

Then we have

$$\mathbf{Pr}[X \leqslant (1+\delta)\frac{S}{\tilde{a}}] \quad \geqslant \quad \mathbf{Pr}[X \leqslant (1+\delta)\mu] \quad \geqslant \quad 1 - \left(\frac{e^\delta}{(1+\delta)^{1+\delta}}\right)^\mu \tag{13}$$

We use the Chernoff bound with $(1+\delta) = e$ and arrive at the following

$$\mathbf{Pr}[X \leqslant e\frac{S}{\tilde{a}}] \quad \geqslant \quad 1 - \exp(-\mu) = 1 - \exp(-S') \tag{14}$$

So the claim holds.

□

S6 Descriptions of 38 RNA-Seq Datasets

See Table S2.

Table S2. Descriptions of 38 RNA-Seq Datasets from *NCBI Sequence Read Archive*.

SRA Study ID	Sample Size	Description
SRP000228	6	RNASeq expression profiling of ENCODE project common cell lines.
SRP000302	6	15 diverse human tissue and cell line transcriptomes on the basis of deep sequencing of complementary DNA fragments.
SRP000403	12	We used a total of 251,933,381 short-read sequence tags generated from various types of transcriptome analyses in order to characterize 6039 iTSCs.
SRP000626	6	We carried out the first analysis of alternative splicing complexity in human tissues using mRNA-Seq data. New splice junctions were detected in 20% of multiexon genes, many of which are tissue specific. By combining mRNA-Seq and EST-cDNA sequence data, we estimate that transcripts from 95% of multiexon genes undergo alternative splicing and that there are 100,000 intermediate- to high-abundance alternative splicing events in major human tissues. From a comparison with quantitative alternative splicing microarray profiling data, we also show that mRNA-Seq data provide reliable measurements for exon inclusion levels. 32-nucleotide sequence reads from six human tissues including brain, cerebral cortex, heart, liver, lung and skeletal muscle.
SRP000698	15	deep human transcriptome-wide resequencing (RNA-seq) for ASE analysis.
SRP000727	16	15 diverse human tissue and cell line transcriptomes based on deep sequencing of cDNA fragments.
SRP000931	14	Paired end sequencing of cDNA isolated from individual melanoma samples to identify genetic aberrations that may play a role in melanoma genesis. Libraries sequenced using the Illumina sequencing platform.
SRP001119	6	RNA-sequencing (RNA-seq) to delineate the per-base expression and splicing developmental changes in the human temporal lobe.
SRP001540	161	RNA-Seq in 69 lymphoblastoid cell lines from multiple Yoruban HapMap individuals in at least two replicate lanes per individual.

Continued on next page

Table S2 – continued from previous page

SRA Study ID	Sample Size	Description
SRP001558	12	Examination of gene expression levels in livers from three primate species (human, chimpanzee, and rhesus macaque), using 3 male and 3 female samples from each species.
SRP001563	41	In this genetic of gene expression study, we used a large sample to search the genome for polymorphic regulators that influence gene expression, and followed up the results with deep sequencing of transcriptomes and molecular analyses.
SRP001847	8	A study to evaluate the effect of flowcell and library preparation on the results of transcriptome sequencing using the Illumina Genome Analyzer. The study has two components. (1) Two different samples were spread over 2 different flowcells. We obtained data with and without using the phiX control lane. (2) One sample was prepared in four different library preparations that were spread over 2 different flowcells.
SRP001851	8	In this study the transcriptome of a chinese hamster ovary (CHO) cell line has been sequenced using the Illumina mRNAseq protocol. Cells which were treated with two different concentrations (0.5mM and 1.0mM) of sodium butyrate on day 4.5 of the experiment were compared to a control group which were grown without butyrate addition on day 4.5. mRNA samples were taken on day 0, day 6 and day 8 of the study.
SRP001998	8	RNA-seq profiles of TAp73alpha, TAp73beta and p53 stably transfected in human osteosarcoma Saos cells.
SRP002079	20	Characterization of changes in the transcriptome profiles during early stages of human neural differentiation from H1 hESCs.
SRP002126	14	Genome-wide comparison of Pol II and NF-KappaB binding in ten individuals. RNA-seq study with no treatment.
SRP002128	10	Human variation in PolII and NF-KappaB binding (RNA-seq study with TNF-alpha induced).
SRP002274	6	Apply the Illumina next generaton sequencing technology to obtain 22 millions of 50-bp paired-end reads Overall Design: High-throughput RNA-seq in human brain tissues.
SRP002543	27	Global 5'-phosphate-dependent RACE in WT, Ago2-KO and Drosha-excised mouse ES cells and human 293S cells.
SRP002605	12	Examine ribosome footprints and mRNA abundance of HeLa cells transfected with miR-1 or miR-155, versus mock-transfected cells, at two different time points post-transfection.

Continued on next page

Table S2 – continued from previous page

SRA Study ID	Sample Size	Description
SRP002628	30	We sequenced the transcriptome (polyA+) of 20 prostate cancer tumors and 10 matched normal tissues using Illumina GAII platform. Then we used bioinformatic approaches to identify prostate cancer specific aberrations which include gene fusion, alternative splicing, somatic mutation, etc.
SRP003186	7	We applied paired-end RNA-seq to characterize 24 novel and 3 previously known fusion genes in breast cancer cells.
SRP003497	28	This data was produced by the Wold lab at Caltech as part of the ENCODE Project.
SRP003611	8	Prostate adenocarcinoma and matched adjacent normal samples were profiled by deep transcriptional sequencing to analyze transcription-induced chimeras and gene fusions. Reference samples from the MAQC and brain and universal reference libraries were also sequenced.
SRP003672	12	We have used deep sequencing to explore the repertoire of both poly(A)+ and poly(A)- RNAs from two standard cell lines, HeLa cells and human embryonic stem cell (hESC) H9 cells.
SRP003767	7	HEK-293T cells expressing KSHV SOX (ORF37), a mutant SOX-P176S or MHV-68 muSOX (ORF37) were subjected to RNAseq analysis.
SRP004776	17	Profiling of Brain, Liver, K562 and Ewing Tumors RNA populations using a novel sequencing approach relying on Helicos single molecule sequencing.
SRP004879	6	Illumina RNA-Seq analysis to survey transcriptome profiles from total brain, frontal and temporal lobe of healthy and AD post-mortem tissue.
SRP004903	8	Examination exon/gene expression of liver and muscle in quadraplicates using both the array technology and RNA-Seq.
SRP005242	21	The second wave of next generation sequencing technologies, referred to as single-molecule sequencing (SMS), carries the promise of profiling samples directly without employing polymerase chain reaction steps used by amplification-based sequencing (AS) methods. To examine the merits of both technologies, we examine mRNA sequencing results from single-molecule and amplification-based sequencing in a set of human cancer cell lines and tissues.
SRP005408	31	Using Illumina's Genome Analyzer, we profiled gene expression in postmortem hippocampus using RNAseq. Comparison was performed among different groups of addicted human samples (Cocaine Overdose, Excited Delirium, Alcohol Abuse and Control).

Continued on next page

Table S2 – continued from previous page

SRA Study ID	Sample Size	Description
SRP005601	8	We used deep sequencinge technology to profile the transcriptome, gene copy number, and CpG island methylation status simultaneously in eight commonly used breast cell lines to develop a model for how these genomic features are integrated in estrogen receptor positive (ER+) and negative breast cancer.
SRP006040	69	Studying vairous sources of biases in RNAseq experiments using Helicos single molecule sequencing.
SRP006731	11	Next generation Sequencing for Gene expression using the RNA-Seq methodology from LNCaP and PrEC cell lines.
SRP007359	26	We describe PolyA-Seq, a strand-specific method for high-throughput sequencing of the 3' ends of polyadenylated transcripts.
ERP000087	11	We report an alternative approach to transcriptome sequencing for the Illumina Genome Analyzer, in which the reverse transcription reaction takes place on the flowcell.
ERP000101	73	RNA-sequencing of 60 HapMap CEU individuals.
ERP000546	48	Transcription profiling by high throughput sequencing of individual and mixture of 16 human tissues RNA.

S7 Signal extraction procedure of the Encyclopedia of DNA Elements (ENCODE) data

Recently, in the ENCODE production phase (September 2007 $\sim$ present) [16], there are 191 ENCODE genome-wide tables for ChIP-seq. To perform enrichment analysis, we integrated these ChIP-seq samples in Genome-wide ENCODE data including chromatin modification, TF binding, Methylation, and chromatin accessibility. UCSC database provides the peak tables for these ChIP-Seq data.

However, different data types have different criterion for the significance. We used both top N and threshold condition to select peaks as follows:

1. If the table is Methylation-Seq data, select the peaks with score > 0.6;
2. Else if the peaks have p-value, select the peaks that are in top 20K and their p-values are $<$ 1E-4;
3. Otherwise, select the peaks that are in top 20K and their signal values > 1.

Then for each table, we selected the target genes whose transcription start site is nearby the peaks within 5000 base pairs.

References

[1] Zhang T (2010) Analysis of multi-stage convex relaxation for sparse regularization. J Mach Learn Res 11: 1081–1107.

[2] Rockafellar RT (1970) Convex Analysis. Princeton, NJ: Princeton University Press.

[3] Yuille AL, Rangarajan A (2003) The concave-convex procedure. Neural Comput 15: 915–936.

[4] Fujita PA, Rhead B, Zweig AS, Hinrichs AS, Karolchik D, et al. (2011) The UCSC genome browser database: update 2011. Nucleic Acids Research 39: D876–882.

[5] Trapnell C, Pachter L, Salzberg SL (2009) TopHat: discovering splice junctions with RNA-Seq. Bioinformatics 25: 1105 –1111.

[6] Trapnell C, Williams BA, Pertea G, Mortazavi A, Kwan G, et al. (2010) Transcript assembly and quantification by RNA-Seq reveals unannotated transcripts and isoform switching during cell differentiation. Nature Biotechnology 28: 511–515.

[7] Jiang H, Wong WH (2009) Statistical inferences for isoform expression in RNA-Seq. Bioinformatics 25: 1026–1032.

[8] Zhou X, Kao M, Huang H, Wong A, Nunez-Iglesias J, et al. (2005) Functional annotation and network reconstruction through cross-platform integration of microarray data. Nat Biotechnol 23: 238–243.

[9] Anderson TW (2003) An introduction to multivariate statistical analysis. Hoboken, NJ: Wiley-Interscience, 3 edition.

[10] Xu M, Kao MCJ, Nunez-Iglesias J, Nevins JR, West M, et al. (2008) An integrative approach to characterize disease-specific pathways and their coordination: A case study in cancer. BMC Genomics 9: S12.

[11] Li W, Liu CC, Zhang T, Li H, Waterman MS, et al. (2011) Integrative analysis of many weighted co-expression networks using tensor computation. PLoS Comput Biol 7: e1001106.

[12] Tsay AA, Lovejoy WS, Karger DR (1999) Random sampling in cut, flow, and network design problems. Math of Oper Res 24: 383–413.

[13] Achlioptas D, McSherry F (2007) Fast computation of low-rank matrix approximations. J ACM 54: 9.

[14] Arora S, Hazan E, Kale S (2006) Approximation, Randomization, and Combinatorial Optimization. Algorithms and Techniques, Berlin Heidelberg: Springer-Verlag, chapter A Fast Random Sampling Algorithm for Sparsifying Matrices. pp. 272–279.

[15] Motwani R, Raghavan P (1995) Randomized Algorithms. Cambridge University Press.

[16] Thomas DJ, Rosenbloom KR, Clawson H, Hinrichs AS, Trumbower H, et al. (2007) The ENCODE project at UC santa cruz. Nucleic Acids Res 35: D663–D667.
